# A propensity score‐matched comparison of neoadjuvant chemoradiotherapy with cisplatin‐5FU and carboplatin–paclitaxel in locally advanced esophageal squamous cell carcinoma: A Turkish oncology group study

**DOI:** 10.1002/cam4.70002

**Published:** 2024-07-18

**Authors:** Fatih Gürler, Fatih Tay, Zehra Sucuoğlu İşleyen, Tahir Yerlikaya, Engin Hendem, Selin Aktürk Esen, Osman Sütçüoğlu, Deniz Işık, Büşra Niğdelioğlu, Miraç Özen, Elif Şahin, Teoman Şakalar, Nilay Şengül Samancı, Özkan Alan, İlhan Hacıbekiroğlu, Efnan Algın, Melek Karakurt Yılmaz, Hacı Mehmet Türk, Berna Öksüzoğlu, Aydın Yavuz, Osman Yüksel, Hüseyin Bora, Ozan Yazıcı, Ahmet Özet, Nuriye Özdemir

**Affiliations:** ^1^ Department of Medical Oncology Gazi University Faculty of Medicine Ankara Turkey; ^2^ Dr Abdurrahman Yurtaslan Ankara Oncology Teaching and Research Hospital, University of Health Sciences Ankara Turkey; ^3^ Department of Medical Oncology Bezmi Alem Vakif University İstanbul Turkey; ^4^ Department of Medical Oncology Antalya Teaching and Research Hospital Antalya Turkey; ^5^ Department of Medical Oncology Necmettin Erbakan University Faculty of Medicine Konya Turkey; ^6^ Department of Medical Oncology University of Health Sciences, Ankara City Hospital Ankara Turkey; ^7^ Department of Medical Oncology Medical Park Kocaeli Hospital Kocaeli Turkey; ^8^ Department of Medical Oncology Ümraniye Teaching and Research Hospital İstanbul Turkey; ^9^ Department of Medical Oncology Sakarya University Faculty of Medicine Sakarya Turkey; ^10^ Department of Medical Oncology Kocaeli University Faculty of Medicine Kocaeli Turkey; ^11^ Department of Medical Oncology Kahramanmaraş Teaching and Research Hospital Kahramanmaraş Turkey; ^12^ Department of Medical Oncology İstanbul Teaching and Research Hospital İstanbul Turkey; ^13^ Department of Medical Oncology Tekirdağ City Hospital Tekirdağ Turkey; ^14^ Department of General Surgery Gazi University Faculty of Medicine Ankara Turkey; ^15^ Department of Radiation Oncology Gazi University Faculty of Medicine Ankara Turkey

**Keywords:** CF, CROSS, esophageal cancer, neoadjuvant

## Abstract

**Background:**

Neoadjuvant treatment is the standard treatment in locally advanced ESCC. However, the optimal chemotherapy regimen is not known.

**Method:**

This is a retrospective observational cohort study conducted with propensity score matching. Patients with resectable ESCC from 13 tertiary centers from Türkiye were screened between January 2011 and December 2021. We compared the efficacy and safety of neoadjuvant chemoradiotherapy with the CF and the CROSS regimens in patients with ESCC.

**Results:**

Three hundred and sixty‐two patients were screened. Patients who received induction chemotherapy (*n* = 72) and CROSS‐ineligible (*n* = 31) were excluded. Two hundred and fifty nine patients received neoadjuvant chemoradiotherapy. After propensity score matching (*n* = 97 in both groups), the mPFS was 18.4 months (95% CI, 9.3–27.4) and 25.7 months (95% CI, 15.6–35.7; *p* = 0.974), and the mOS was 35.2 months (95% CI, 18.9–51.5) and 39.6 months (95% CI 20.1–59.2; *p* = 0.534), in the CF and the CROSS groups, respectively. There was no difference between subgroups regarding PFS and OS. Compared with the CF group, the CROSS group had a higher incidence of neutropenia (34.0% vs. 62.9%, *p* < 0.001) and anemia (54.6% vs. 75.3%, *p* = 0.003) in all grades. On the other hand, there was no significant difference in grade 3–4 anemia, grade 3–4 neutropenia, and febrile neutropenia between groups. There were more dose reductions and dose delays in the CROSS group than in the CF group (11.3% vs. 3.1%, *p* = 0.026 and 34.0% vs. 17.5%, *p* = 0.009, respectively). The resection rate was 52.6% in the CF‐RT and 35.1% in the CROSS groups (*p* = 0.014).

**Conclusion:**

Favorable PFS and pCR rates and a comparable OS were obtained with the CROSS regimen over the CF regimen as neoadjuvant chemoradiotherapy in patients with ESCC.

## 1 INTRODUCTION

Esophageal cancer is the eleventh most common cancer and the seventh leading cause of cancer‐related mortality, according to GLOBOCAN 2022.^1^ Although it has distinct histological subtypes, multidisciplinary treatment approaches improved survival outcomes. Neoadjuvant therapy is the standard treatment in locally advanced esophageal cancer.^2^ Neoadjuvant chemoradiotherapy with cisplatin‐5 Fluorouracil (CF) based regimens plus surgery was the widely used therapy for esophageal cancer for decades.^3^, ^4^, ^5^ In the CALGB 9781 trial, it was shown that trimodal therapy (chemoradiotherapy with CF plus surgery) improved overall survival (OS) over surgery alone (4.48 years vs. 1.79 years, *p* = 0.002, respectively). Furthermore, the pathological complete response (pCR) was reported as 40% in the CALGB 9781 trial. After that, CALGB 9781 trimodal therapy was adopted as a standard of care in locally advanced esophageal cancer.^6^ On the other hand, in the CROSS trial, trimodal therapy (chemoradiotherapy with carboplatin plus paclitaxel plus surgery) demonstrated an improved OS (48.6 months vs. 24.0 months, HR 0.68, *p* = 0.003, respectively) over surgery alone with similar surgical outcomes. In addition, the pCR rate was reported as 29% in the trimodal therapy cohort. Although most of the patients had adenocarcinoma histopathology in the CROSS trial, in the subgroup analysis, it was shown that the pCR rate was 49% and the median OS (mOS) was 81.6 months (95% CI 47.2–116.0) in patients with esophageal squamous cell carcinoma (ESCC).^7^, ^8^

Even though the CROSS regimen is a more commonly used neoadjuvant approach than the CF regimen, no randomized trial compares neoadjuvant therapy with CF and CROSS regimens, especially in patients with ESCC. There is no clear consensus on the optimal chemotherapy regimen in a neoadjuvant chemoradiotherapy setting. There are a few retrospective studies with a limited number of patients.^9^, ^10^, ^11^, ^12^ Therefore, in this study, we aimed to compare the efficacy and the safety of neoadjuvant chemoradiotherapy with the CF and the CROSS regimens in patients with ESCC as a propensity score‐matched cohort retrospectively.

## 2 MATERIALS AND METHODS

### 2.1 Study population

**FIGURE 1:**
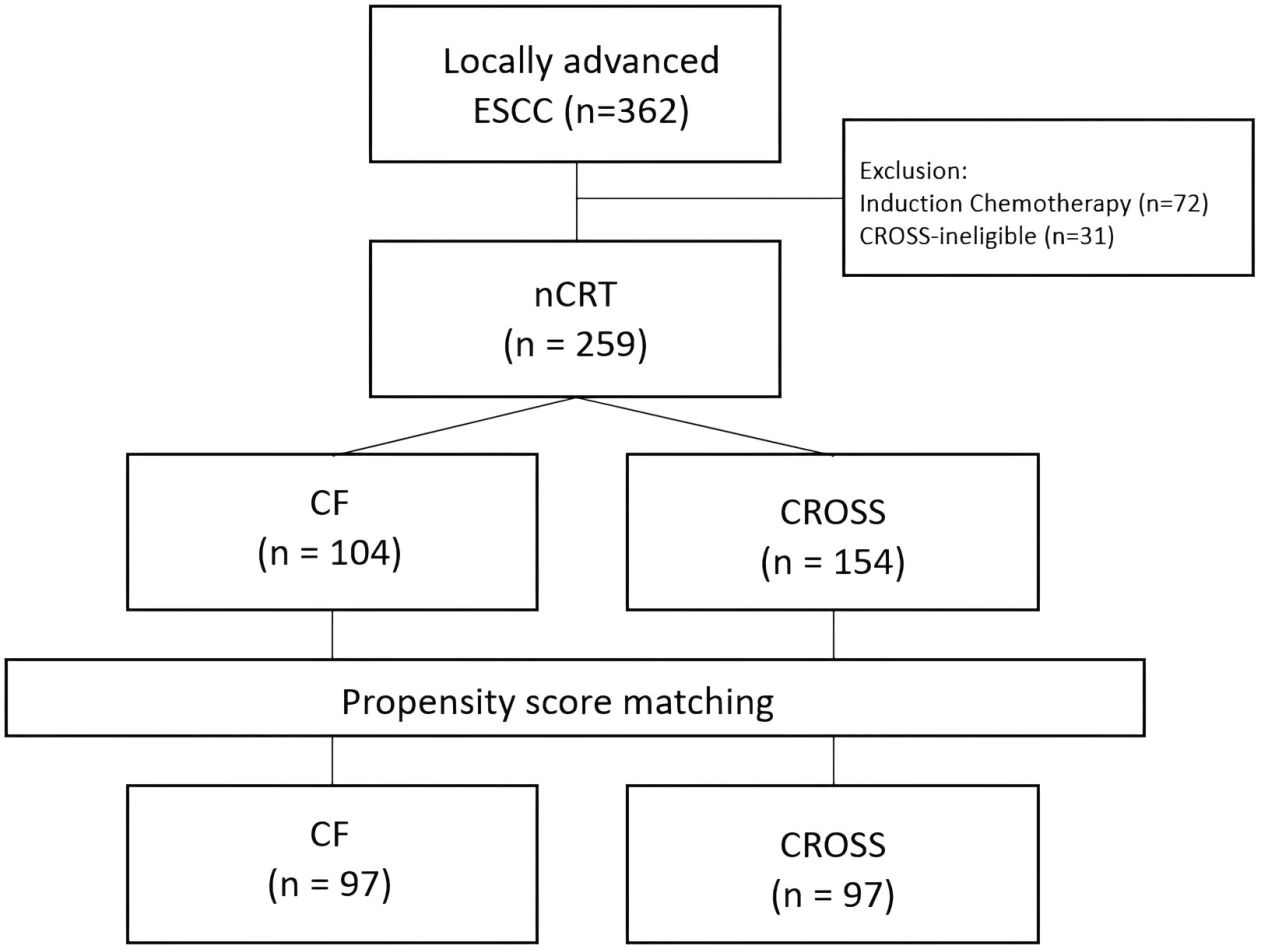
Flowchart of patient inclusion and exclusion and number of patients.

This is a retrospective cohort observational study conducted with propensity score matching. Patients with resectable ESCC from 13 tertiary centers in Türkiye were screened between January 2011 and December 2021. Inclusion criteria were defined as being 18 years of age or older, having SCC histopathology, resectable tumor with CROSS eligibility (clinic stage T1N1 or T2‐3, N0‐1), and received neoadjuvant chemoradiotherapy either with CROSS regimen or cisplatin plus 5‐fluorouracil (CF). Exclusion criteria were secondary malignancy, mixed histopathology, induction chemotherapy, or CROSS ineligibility. The data were retrieved from patients' medical records (Figure 1).

Progression‐free survival (PFS) was the time between the diagnosis and disease progression or death (in months). The Response Evaluation Criteria in Solid Tumors (RECIST) (version 1.1) criteria were used to define progression. The OS was the time between the diagnosis and death (in months). The OS in resected patients of the propensity score‐matched cohort was presented with rOS. The disease‐free survival (DFS) was the time between surgery and disease progression or death (in months). The patients who lost follow‐up were censored. The National Cancer Institute Common Terminology Criteria for Adverse Events, Version 4.0, was used to grade adverse events.

### 2.2 Treatment schedules

In Türkiye, the CROSS regimen was adopted in 2014. Before that, only the CF regimen was used concurrently with radiotherapy. After 2014, both regimens have been widely used. The CF regimen was administered as cisplatin 100 mg/m^2^/day iv on day 1, and 5‐FU 1000 mg/m^2^/day iv on days 1–5 and 22–26 concurrent with radiotherapy (total 54 Gy, 1.8 Gy/fr). The CROSS regimen was administered as carboplatin (AUC2) iv on day 1 and paclitaxel 50 mg/m^2^/day on day 1 weekly for 5 weeks concurrently with radiotherapy (total 41.4 Gy, 1.8 Gy/fr). Three‐dimensional conformal or intensity‐modulated radiotherapy techniques were used depending on the approach of participant clinics. All the prescribed radiotherapy regimes were completed. When no problems were detected regarding surgical tolerability after neoadjuvant treatment in the two groups, surgery was performed in 4–6 weeks. In surgery, transhiatal and transthoracic approaches and 2‐field or 3‐field dissection were performed. The choice of surgical method depended on the location and stage of the tumor, the length of the tumorous esophageal segment, age, comorbidities, and previous gastrointestinal operations. Right thoracotomy, gastric pull‐up for reconstruction, stapler anastomosis for thoracic anastomosis, and hand anastomosis for cervical anastomosis were preferred. No adjuvant treatment was given to patients.

### 2.3 Statistical analysis

The primary outcomes were PFS and OS. The secondary outcome was pCR.

Descriptive statistical analyses were conducted to illustrate the distribution and homogeneity of the variables. Continuous variables were reported using the median (interquartile range: IQR); categorical variables were reported using Pearson's Chi‐squared or Fisher's Exact tests. Survival curves were created by the Kaplan–Meier method and compared with the log‐rank test. Univariate and multivariate analyses were conducted to show the effects of variables on progression and death in resected patients of the propensity score‐matched cohort.

Propensity score matching was performed with a logistic regression model for variables of elderly, sex, ECOG PS, tumor level, clinical T stage, and clinical N stage. Patients were matched (1:1) according to nearest neighbor matching without replacement. A caliper of 0.15 was performed as the maximum difference tolerated. Checking the goodness of balancing propensity was reported with standardized mean differences. The Cox proportional regression analysis was conducted to estimate the hazard ratio (HR), 95% confidence interval (CI), and *p*‐value and presented with a forest plot. The proportional hazard assumption for Cox regression was evaluated with visual presentations of log‐(log(survival)) and assessment of the time‐dependent Cox model (time as a linear variable). Statistical analysis was performed with S.P.S.S. version 26.0 (S.P.S.S. Inc., Chicago, IL, U.S.A.). All tests were bidirectional, and the *p* < 0.05 was accepted as significant.

## 3 RESULTS

### 3.1 Patient characteristics

Three hundred and sixty‐two patients were screened. Patients who received induction chemotherapy (*n* = 72) and CROSS‐ineligible (*n* = 31) were excluded. Two hundred and fifty‐nine patients received neoadjuvant chemoradiotherapy. Before propensity score matching, 40.5% (*n* = 104) of the patients received the CF, and 59.5% (*n* = 155) received the CROSS regimen. ECOG PS and clinical T stage were not well balanced between unmatched cohorts. After matching, 97 patients in each group with equally distributed characteristics were included in the analysis. The baseline characteristics of the unmatched and matched cohorts are shown in Table 1.

**TABLE 1:** The baseline characteristics of patients before and after propensity score matching.

Variable	Before matching				After matching				
	CF	CROSS	Total	*p* Value	CF	CROSS	Total	*p* Value	*d*
Number of patients, *n* (%)	104 (40.5)	155 (59.5)	259	–	97 (50.0)	97 (50.0)	194	–	–
Median age, *years* (IQR)	54 (48–64)	57 (49–64)	56 (48–64)	0.091	54 (48–64)	58 (50–65)	57 (49–65)	0.312	–
Elderly, *n* (%)									
<65 years old	81 (77.9)	117 (75.5)	198 (76.4)	0.655	74 (76.3)	71 (73.2)	145 (74.7)	0.620	0.02
≥65 years old	23 (22.1)	38 (24.5)	61 (23.6)		23 (23.7)	26 (26.8)	49 (25.3)		
Sex, *n* (%)									
Female	44 (42.3)	73 (47.1)	117 (45.2)	0.448	41 (42.3)	47 (48.5)	88 (45.4)	0.387	0.03
Male	60 (57.7)	82 (52.9)	142 (54.8)		56 (57.7)	50 (51.7)	106 (54.6)		
ECOG PS, *n* (%)									
0–1	**96 (92.3)**	**152 (98.1)**	**248 (95.7)**	**0.024**	91 (93.8)	94 (96.9)	185 (95.4)	0.306	0.04
2	**8 (7.7)**	**3 (1.9)**	**11 (4.3)**		6 (6.2)	3 (3.1)	9 (4.6)		
Tumor level, *n* (%)									
Upper	22 (21.2)	40 (25.8)	62 (23.9)	0.567	20 (20.6)	25 (25.8)	45 (23.2)	0.167	0.01
Middle	50 (48.1)	65 (41.9)	115 (44.4)		47 (48.5)	34 (35.1)	81 (41.7)		
Lower	32 (30.8)	50 (32.3)	82 (31.7)		30 (30.9)	38 (39.2)	68 (35.1)		
Clinical T stage, *n* (%)									
T1	**9 (8.7)**	**1 (0.6)**	**10 (3.8)**	**0.001**	4 (4.1)	3 (3.1)	7 (3.6)	0.576	0.03
T2	**20 (19.2)**	**47 (30.3)**	**67 (25.9)**		20 (20.6)	26 (26.8)	46 (23.7)		
T3	**75 (72.1)**	**107 (69.0)**	**182 (70.2)**		73 (75.3)	68 (70.1)	141 (72.7)		
Clinical N stage, *n* (%)									
N0	42 (40.4)	51 (32.9)	93 (35.9)	0.219	42 (43.3)	37 (38.1)	79 (40.7)	0.465	0.03
N1	62 (59.6)	104 (67.1)	166 (64.1)		55 (56.7)	60 (61.9)	115 (59.3)		

*Note*: *d*, Standardized mean difference. The statistically significant values are presented in bold.

### 3.2 Survival and subgroup analyses in the PSM cohort

**FIGURE 2:**
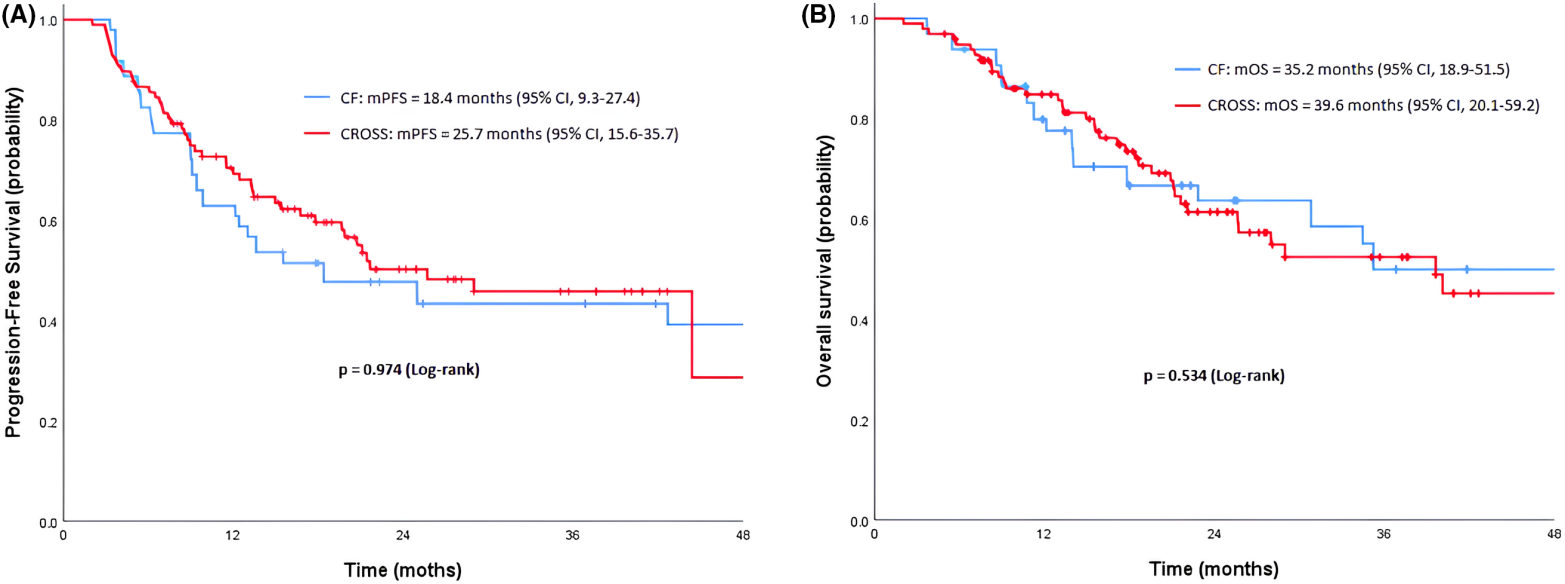
Kaplan–Meier curves of overall survival (OS) (A) and progression‐free survival (PFS) (B) in PSM cohort.

**FIGURE 3:**
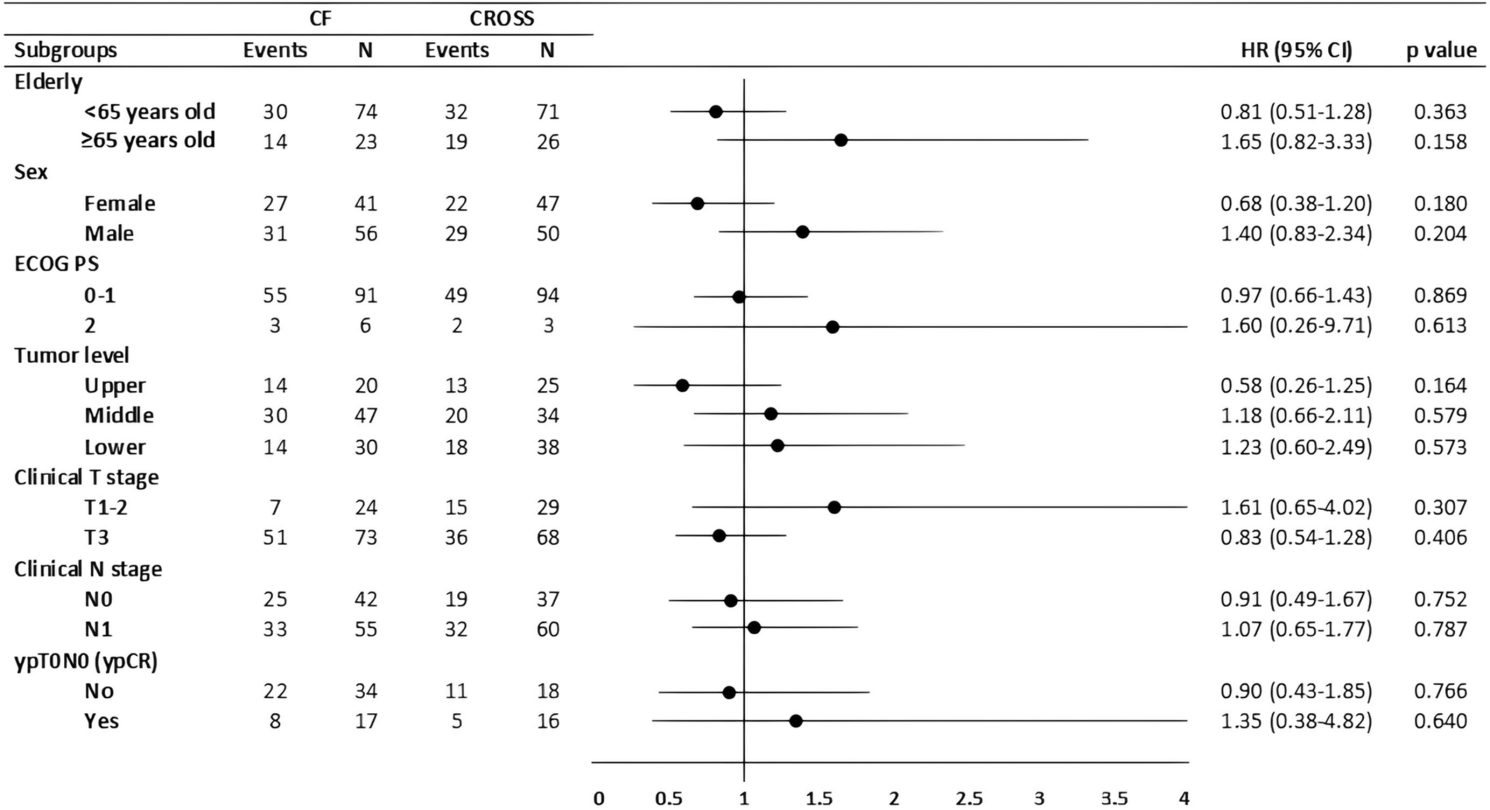
Subgroup analysis of progression‐free survival (PFS) in the propensity score‐matched (PSM) cohort.

The median follow‐up in the whole cohort was 19.8 months (IQR: 11.2–35.3). The median follow‐up was longer in the CF group than in the CROSS group (21.7 months vs. 18.9 months, *p* = 0.027; Table 2). The mPFS was 18.4 months (95% CI, 9.3–27.4) in the CF and 25.7 months (95% CI, 15.6–35.7) in the CROSS groups, and the difference was not significant (HR 0.99; 95% CI 0.68–1.46; *p* = 0.974; Figure 2A). Subgroup analyses for PFS in the PSM cohort showed no significant difference between the CF and the CROSS groups (Figure 3).

**TABLE 2:** Responses to neoadjuvant treatment and surgical outcomes in the propensity score‐matched cohort.

Variable	CF	CROSS	*p* Value
Median follow‐up, months, median (IQR)	**21.7 (11.9–41.8)**	**18.9 (10.8–27.7)**	**0.027**
Surgery, *n* (%)			
No	**46 (47.4)**	**63 (64.9)**	**0.014**
Yes	**51 (52.6)**	**34 (35.1)**	
Time from diagnosis to surgery, months, median (IQR)	5.3 (4.4–5.9)	4.8 (4.1–5.4)	0.269
Reason of no surgery, *n* (%)			
Disease progression	17 (37.0)	15 (23.8)	0.062
Poor performance status	0 (0.0)	6 (9.5)	
Physician choice	6 (13.0)	14 (22.2)	
Patient refusal	23 (50.0)	28 (44.4)	
Resection margin, *n* (%)			
R0	**45 (88.2)**	**34 (100.0)**	**0.038**
R1	**6 (11.8)**	**0 (0.0)**	
R2	**0 (0.0)**	**0 (0.0)**	
ypT stage, *n* (%)			
ypT0	21 (41.2)	18 (52.9)	0.239
ypT1	10 (19.6)	9 (26.5)	
ypT2	3 (5.9)	(0.0)	
ypT3	17 (33.3)	7 (20.6)	
ypN stage, *n* (%)			
ypN0	**20 (39.2)**	**24 (70.6)**	**0.014**
ypN1	**31 (60.8)**	**10 (29.4)**	
ypT0N0 (ypCR), *n* (%)			
No	34 (66.7)	18 (52.9)	0.203
Yes	17 (33.3)	16 (47.1)	
Recurrence patterns in R0‐resected cases, *n* (%)			
Locoregional recurrence	5 (20.8)	3 (18.8)	0.502
Distant metastasis	14 (58.3)	7 (43.8)	
Locoregional recurrence + distant metastasis	5 (20.8)	6 (37.5)	

*Note*: The statistically significant values are presented in bold.

**FIGURE 4:**
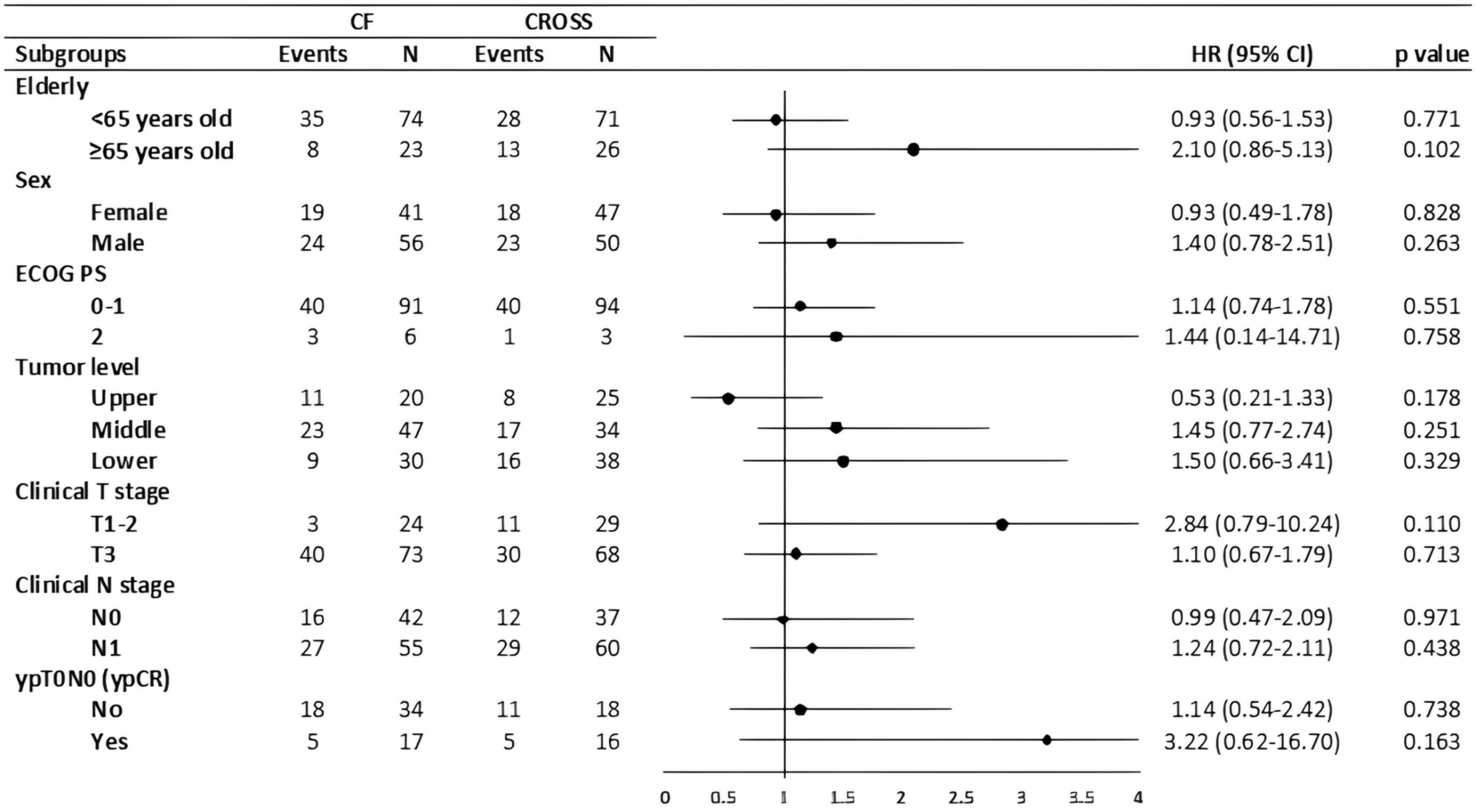
Subgroup analysis of overall survival (OS) in the propensity score‐matched (PSM) cohort.

The mOS was 35.2 months (95% CI, 18.9–51.5) in the CF and 39.6 months (95% CI 20.1–59.2) in the CROSS groups, and the difference was not significant (HR 1.15; 95% CI 0.74–1.77; *p* = 0.534; Figure 2B). Subgroup analyses for OS in the PSM cohort showed no significant difference between the CF and the CROSS groups (Figure 4).

### 3.3 Neoadjuvant treatment and surgical outcomes

The resection rate in the CF group was significantly higher than in the CROSS group (52.6% vs. 35.1%, respectively, and *p* = 0.014). Patient refusal was the most common reason for no surgery in both groups. While all of the resections in the CROSS group were R0, 88.2% were R0, with the remaining R1 in the CF group (*p* = 0.038). In both groups, most patients had a pathological complete response in the primary tumor (ypT0). On the other hand, the CROSS group had significantly higher rates of pathological response in lymph nodes (ypN0) than the CF group (70.6% vs. 39.2%, respectively, and *p* = 0.014). A statistically nonsignificantly higher pathological complete response rate (ypT0N0, pCR) was observed in the CROSS group than in the CF group (47.1% vs. 33.3%, respectively, and *p* = 0.203). Most patients in both groups had distant recurrence, and there was no difference in recurrence patterns. Responses to neoadjuvant treatment and surgical outcomes in the propensity score‐matched (PSM) cohort are shown in Table 2.

In surgically resected patients of the PSM cohort, the mDFS was 31.8 months (95% CI 9.8–53.8) in the CF and 24.2 months (95% CI 21.2–26.9) in the CROSS groups (*p* = 0.654) (Figure S1A). In the univariate analyses to estimate variables' effects on recurrence, it was observed that the ypT0N0 variable was associated with a longer DFS (HR 0.30; 95% CI 0.15–0.60, *p* = 0.001; Table S1). Since it was the sole variable in univariate analyses, no further multivariate analysis was carried out. In surgically resected patients of the PSM cohort, the mrOS was 53.4 months (95% CI 32.7–74.0) in the CF and 40.1 months (95% CI 30.9–49.5) in the CROSS groups (*p* = 0.545; Figure S1B). In the univariate analyses to estimate variables' effects on death, it was found that the clinical N‐positivity variable was associated with a shorter OS, and the ypT0N0 variable was associated with a longer OS. These effects were also obtained in the multivariable analysis (HR 1.94; 95% CI 1.01–3.73; *p* = 0.049 for clinical N‐positivity variable and HR 0.47; 95% CI 0.23–0.96; *p* = 0.039 for the ypT0N0 variable; Table S2). Local and distant recurrence‐free survivals in surgically resected patients of the PSM cohort are shown in Figure S2.

### 3.4 Safety

Anemia was the most common adverse event. Compared with the CF group, the CROSS group had a higher incidence of neutropenia (34.0% vs. 62.9%, *p* < 0.001) and anemia (54.6% vs. 75.3%, *p* = 0.003) in all grades. On the other hand, there was no significant difference in grade 3–4 anemia, grade 3–4 neutropenia, and febrile neutropenia between groups. There were more dose reductions and dose delays in the CROSS group than in the CF group (11.3% vs. 3.1%, *p* = 0.026 and 34.0% vs. 17.5%, *p* = 0.009, respectively; Table 3).

**TABLE 3:** Safety data and treatment compliance of chemoradiotherapy in the propensity score‐matched cohort.

Variable, *n* (%)	CF		CROSS		*p* Value	
	Any grade	Grade 3–4	Any grade	Grade 3–4	Any grade	Grade 3–4
Neutropenia	**33 (34.0)**	11 (11.3)	**61 (62.9)**	13 (13.4)	**<0.001**	0.663
Anemia	**53 (54.6)**	6 (6.2)	**73 (75.3)**	2 (2.1)	**0.003**	0.149
Thrombocytopenia	31 (32.0)	6 (6.2)	29 (29.9)	3 (3.1)	0.756	0.306
Liver toxicity	17 (17.5)	0 (0.0)	21 (21.6)	2 (2.1)	0.469	0.155
Renal toxicity	9 (9.3)	0 (0.0)	8 (8.2)	0 (0.0)	0.800	–
Other adverse events	19 (19.6)	5 (5.2)	10 (10.3)	1 (1.0)	0.070	0.097
Febrile neutropenia	11 (11.3)		6 (6.2)		0.204	
≥1 dose reduction	**3 (3.1)**		**11 (11.3)**		**0.026**	
≥1 dose delay	**17 (17.5)**		**33 (34.0)**		**0.009**	
Treatment cessation	3 (3.1)		6 (6.2)		0.306	

*Note*: The statistically significant values are presented in bold.

## 4 DISCUSSION

In the present study, we retrospectively compared the efficacy and safety of trimodal therapy with the CROSS and CF regimens in patients with esophageal squamous cell carcinoma in propensity‐matched cohorts. It was demonstrated that the CROSS regimen had similar PFS, OS pCR compared with the CF regimen.

In the CROSS trial, 23% of the patients (*n* = 41) in the trimodal therapy group had SCC histopathology. It was reported that the mPFS was 74.7 months (95% CI 55.1–94.4), the mOS was 81.6 months (95% CI 47.2–116.0), and the pCR rate was 49%. Although our study's CROSS arm has a pCR rate (47.1%) similar to the original CROSS trial, survival outcomes (the mPFS: 25.7 months, the mOS: 39.6 months) were numerically shorter than the original CROSS trial.^7^, ^8^ A minimal number of patients with ESCC in the CROSS trial might prevent a conclusive indirect comparison with the current study. On the other hand, in the CALGB9781 trial, there were only seven patients with ESCC in the trimodal therapy, so a subgroup analysis was not available.^6^ Toxopeus et al.^13^ demonstrated that even more advanced disease (N2–N3) post‐CROSS real‐life outcomes were comparable with the CROSS trial. Likewise, it was shown that the CROSS regimen was less toxic in geriatric patients with similar outcomes.^14^ Several studies clearly stated that survival outcomes were improved in patients with pCR. Besides, residual nodal disease was more prone to a poorer survival.^15^, ^16^ The pCR rates could be a promising surrogate biomarker for survival outcomes.

There were controversial results regarding the optimal chemoradiotherapeutic agent. Münch et al. compared the CROSS and CF regimens in trimodal neoadjuvant setting in patients with ESCC. Although the number of patients was too small (*n* = 51), the mOS was nonsignificantly higher toward the CF regimen (23.9 vs. 40.1 months, *p* = 0.08). The patients' characteristics and survival outcomes are similar to those in our study.^11^ In a retrospective study by Wong et al., the CROSS and CF regimens were compared in neoadjuvant chemoradiotherapy in patients with ESCC. In the PSM cohort CROSS‐eligible subgroup of the study, it was reported that the CF regimen showed a nonsignificantly improved OS (the mOS was 44.9 vs. 21.6 months, *p* = 0.093, respectively) and a nonsignificantly higher pCR rate over the CROSS regimen (41.7% vs. 33.3%, *p* = 0.448, respectively).^12^ Contrary to the study by Wong et al., our study had a non‐significantly improved survival outcomes trend favoring the CROSS trial. The reason for this discrepancy is not clearly understood. However, there might be a few possible reasons for that. Basal characteristics of the study by Wong et al. and our study have some differences. While 57–58% of the patients had middle esophagus SCC in the study by Wong et al., less than half had middle esophagus SCC in our study. Moreover, resection rates are lower in our study. Approximately three‐fourths of the patients underwent resection in the study by Wong et al. In our study, the resection rate was 52.6% in the CF arm and 35.1% in the CROSS arm. Nearly in half of the patients who did not undergo surgery, the reason was patient refusal. This might be a possible bias. If the patient refuses the surgery, the symptoms might be relieved already. Additionally, there might be potential pCR candidates in this group of patients. Still, it does not explain the difference. This study did not cover chemotherapy‐related adverse events, only surgical complications. Distant recurrence was the most common in our cohort, but in the study by Wong et al., synchronous distant + locoregional recurrence was the most common. The most important reason for that might be that the lower resection rate in our cohort paved the way for an early distant recurrence.

Contrary to retrospective studies, Meta‐analyses showed that taxane‐based neoadjuvant chemoradiotherapy improved survival over platinum‐5‐FU neoadjuvant chemoradiotherapy in patients with ESCC.^17^, ^18^ Our study is consistent with these meta‐analyses favoring the CROSS regimen with similar grade 3–4 adverse events. On the other hand, treatment compliance seemed lower in the CROSS arm.

There is no consensus on a standard definition for locally advanced ESCC. After the CROSS trial, there have been clinics in which more advanced‐stage ESCC patients (T4, N2, or N3) were treated with the CROSS trial in neoadjuvant chemoradiotherapy. Multistation nodal disease or T4 disease is considered more prone to anticipate distant metastasis. This might be one of the reasons why the CROSS trial consisted of non‐bulky disease. Likewise, we focused on CROSS‐eligible patients to carry out a fairer comparison between our study and the CROSS trial. The CF regimen should be administered in an inpatient unit or with a central venous port catheter. On the other hand, the CROSS regimen could be administered in an outpatient unit. This means the administration of therapy favors the CROSS regimen.

The current study has some limitations. The retrospective nature of the study might affect the quality of the data. However, a propensity score matching was conducted to adjust the confounders. Multicenter trials are concerned about surgical quality and perioperative care. This might also be accepted as a limitation. Patients were staged with AJCC TNM 8 staging system to make a standardization. This was also a concern when compared with the former clinical trial staging system. Low resection rates compared with the pivotal trials are another limitation. Especially in the CROSS arm of our study, two‐thirds of the patients did not undergo surgery. Furthermore, patient refusal or physician choice is the sole reason for no surgery in two‐thirds of the patients. This might be accepted as a reason why numerically increased pCR rates did not confer a survival benefit in the CROSS arm compared with the CF arm. In the non‐pCR group, nivolumab could not be administered because of the lack of reimbursement, as recommended in the CheckMate 577 trial.^19^ Finally, the median follow‐up time was longer in the CF group than in the CROSS group. This was an expected result due to the older CF regimen.

In conclusion, in the present study, similar PFS, OS, and pCR rates were obtained with the CROSS regimen compared with the CF regimen as neoadjuvant chemoradiotherapy in patients with ESCC. A head‐to‐head comparison in a randomized phase III clinical trial is warranted.

## AUTHOR CONTRIBUTIONS

**Fatih Gürler:** Conceptualization (lead); data curation (lead); formal analysis (lead); methodology (lead); writing – original draft (lead); writing – review and editing (lead). **Fatih Tay:** Data curation (equal). **Zehra Sucuoğlu İşleyen:** Data curation (equal). **Tahir Yerlikaya:** Data curation (equal). **Engin Hendem:** Data curation (equal). **Selin Aktürk Esen:** Data curation (equal). **Osman Sütçüoğlu:** Data curation (equal). **Deniz Işık:** Data curation (equal). **Büşra Niğdelioğlu:** Data curation (equal). **Miraç Özen:** Data curation (equal). **Elif Şahin:** Data curation (equal). **Teoman Şakalar:** Data curation (equal). **Nilay Şengül Samancı:** Data curation (equal). **Özkan Alan:** Data curation (equal). **İlhan Hacıbekiroğlu:** Data curation (equal). **Efnan Algın:** Data curation (equal). **Melek Karakurt Eryılmaz:** Data curation (equal). **Hacı Mehmet Türk:** Data curation (equal). **Berna Öksüzoğlu:** Data curation (equal). **Aydın Yavuz:** Data curation (equal). **Osman Yüksel:** Data curation (equal). **Hüseyin Bora:** Data curation (equal). **Ozan Yazıcı:** Data curation (equal); supervision (equal); writing – review and editing (equal). **Ahmet Özet:** Data curation (equal); supervision (equal); writing – review and editing (equal). **Nuriye Özdemir:** Data curation (equal); supervision (lead); writing – review and editing (lead).

## FUNDING INFORMATION

None to declare.

## CONFLICT OF INTEREST STATEMENT

The authors declare that they have no conflict of interest.

## ETHICS STATEMENT

Ethical approval was waived by the local Ethics Committee of Gazi University School of Medicine (06.05.2021/2021‐530).

## Supporting information


Appendix S1.

## Data Availability

The datasets generated during and/or analyzed during the current study are available from the corresponding author upon reasonable request.
