# Supplementary material for: A propensity score‐matched comparison of neoadjuvant chemoradiotherapy with cisplatin‐5FU and carboplatin–paclitaxel in locally advanced esophageal squamous cell carcinoma: A Turkish oncology group study
Source: Cancer Med. 2024 Jul 18;13(14):e70002. doi: 10.1002/cam4.70002 (PMC11257995; doi:10.1002/cam4.70002)
Supplement: Supplementary file 1 — Appendix S1. [file CAM4-13-e70002-s001.docx]

| **Table S1. Univariate and Multivariate Cox regression Models to estimate Progression in resected patients of PSM cohort** | | | | | | |
| --- | --- | --- | --- | --- | --- | --- |
|  | **Univariate Analysis** | | | **Multivariate Analysis** | | |
| **Variable** | **HR** | **Cl (95%)** | ***p* value** | **HR** | **Cl (95%)** | ***p* value** |
| Elderly  <65 years old  ≥65 years old | Ref  0.81 | 0.25-2.63 | 0.726 | - | - | - |
| Sex  Female  Male | Ref  1.67 | 0.88-3.17 | 0.120 | - | - | - |
| Tumor level  Upper  Middle  Lower | Ref  0.44  0.37 | 0.16-1.18  0.13-1.03 | 0.102  0.058 | - | - | - |
| Clinical T stage  T1  T2  T3 | Ref  0.51  0.66 | 0.18-1.48  0.25-1.73 | 0.216  0.400 | - | - | - |
| Clinical N stage  N0  N1 | Ref  0.69 | 0.35-1.26 | 0.213 | - | - | - |
| **ypT0N0 (ypCR), *n* (%)**  **No**  **Yes** | **Ref**  **0.30** | **0.15-0.62** | **0.001** | **-** | **-** | **-** |
| Neoadjuvant therapy  CF  CROSS | Ref  0.87 | 0.47-1.62 | 0.658 | - | - | - |

| **Table S2. Univariate and Multivariate Cox regression Models to estimate Death in resected patients of PSM cohort** | | | | | | |
| --- | --- | --- | --- | --- | --- | --- |
|  | **Univariate Analysis** | | | **Multivariate Analysis** | | |
| **Variable** | **HR** | **Cl (95%)** | ***p* value** | **HR** | **Cl (95%)** | ***p* value** |
| Elderly  <65 years old  ≥65 years old | Ref  1.03 | 0.31-3.40 | 0.958 | - | - | - |
| Sex  Female  Male | Ref  1.65 | 0.83-3.30 | 0.153 | - | - | - |
| Tumor level  Upper  Middle  Lower | Ref  0.48  0.49 | 0.16-1.43  0.16-1.53 | 0.188  0.218 | - | - | - |
| Clinical T stage  T1  T2  T3 | Ref  1.21  1.54 | 0.32-4.57  0.46-5.19 | 0.784  0.488 | - | - | - |
| **Clinical N stage**  **N0**  **N1** | **Ref**  **2.07** | **1.07-3.99** | **0.031** | **1.94** | **1.01-3.73** | **0.049** |
| **ypT0N0 (ypCR), *n* (%)**  **No**  **Yes** | **Ref**  **0.44** | **0.22-0.91** | **0.027** | **0.47** | **0.23-0.96** | **0.039** |
| Neoadjuvant therapy  CF  CROSS | Ref  1.21 | 0.64-2.31 | 0.554 | - | - | - |


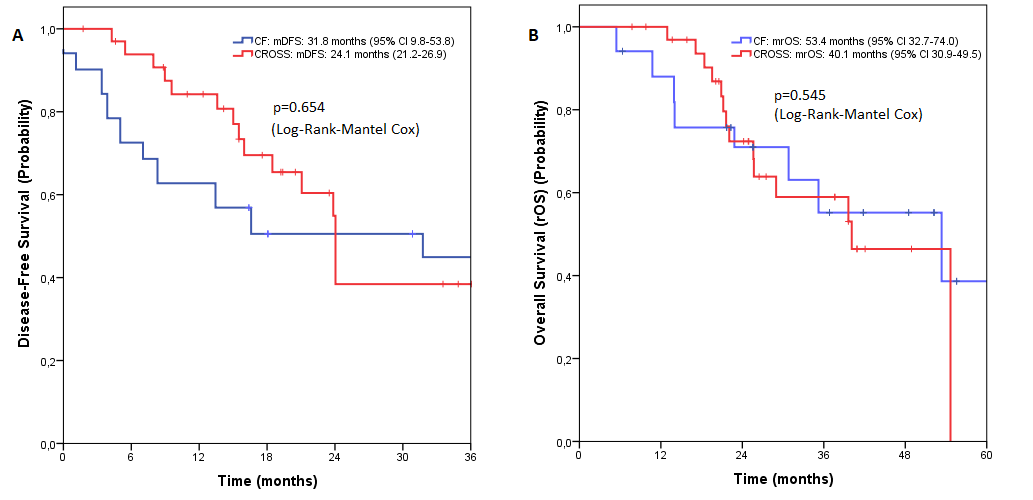


Figure S1: Kaplan-Meier curves of disease-free survival (DFS) (A) and overall survival (rOS) (B) in resected patients of the PSM cohort.


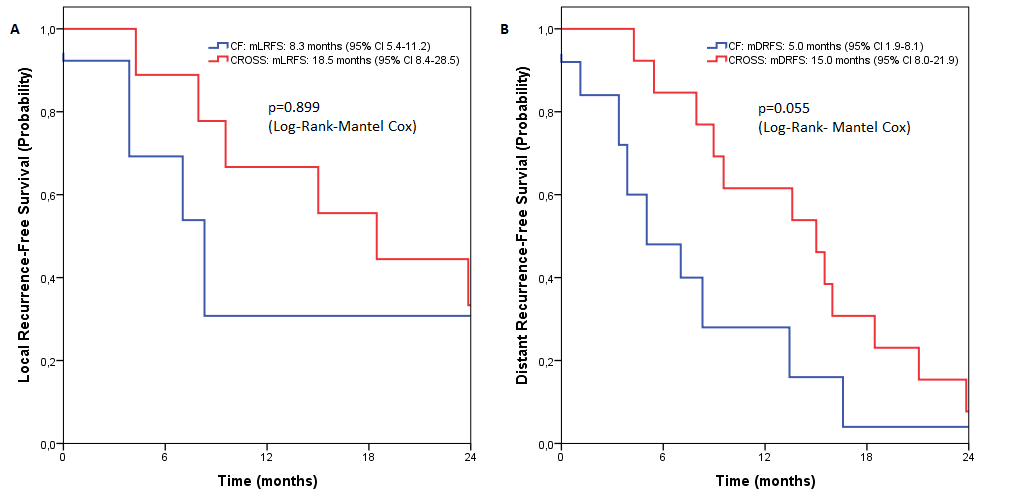


Figure S2: Kaplan-Meier curves of local recurrence-free survival (LRFS) (A) and distant recurrence-free survival (DRFS) (B) in resected patients of the PSM cohort.
